# Strengthening management of non-communicable diseases in primary care, Malawi: A short report

**DOI:** 10.4102/phcfm.v13i1.3053

**Published:** 2021-12-03

**Authors:** Amos Mailosi, Christina Miller, Catherine Hodge, Serah Msimuko

**Affiliations:** 1 School of Public Health and Family Medicine, Faculty of Family Medicine, University of Malawi, College of Medicine, Blantyre, Malawi; 2 Department of Family Medicine, Faculty of Family Medicine, Nkhoma Mission Hospital, Lilongwe, Malawi

**Keywords:** primary care, Malawi, collaboration, non-communicable diseases, resident education, capacity building

## Abstract

Within the community-orientated primary care module for training family physicians at the Kamuzu University of Health Sciences in Malawi, a relationship was formed between Nkhoma Mission Hospital’s Family Medicine Department and the Diamphwe Community Health Centre (HC) to strengthen the continuity of healthcare and capacity team building. The initial focus was on improving the management of hypertension and diabetes in terms of diagnosis, tracking of the patients in a registry and timely referral to secondary care facilities The relationship has received positive support from Diamphwe healthcare workers, which then improved the management of non-communicable diseases and patient care at Diamphwe. It has also shown how family medicine physicians can improve HC capacity through support and mentorship.

## The context

Malawi has attained remarkable improvements in most health indicators over the past 20 years despite having a low doctor-to-patient ratio of 1:50 000. These improvements have been due in large part to a focus on essential service delivery in maternal–child health.^1,2^ Eighty per cent of the country’s population live in rural communities, whereas the majority of doctors work in the country’s four urban central hospitals.^3^ Most rural care in Malawi is provided by clinical officers, who have undergone a 3-year clinical training, and by medical assistants, who have undergone a 2-year clinical training.

Health centres (HCs) are usually staffed by medical assistants, although sometimes they may have a clinical officer or only nurses. These HCs provide basic services to rural communities and refer complicated patients to district hospitals, which should have at least two generalist doctors. District hospitals then refer to central hospitals for specialised care. According to a report from the Ministry of Health, 70% of cases referred for tertiary care could have been managed at primary care or district hospitals.^4^ Challenges in gatekeeping, as well as limitations in resources, time and training, often limit the ability of rural healthcare providers to appropriately address chronic diseases and complicated cases. Of particular concern is the rising burden of non-communicable diseases (NCDs). An estimated 32% of Malawians have hypertension and 6% have diabetes mellitus. These cases are often referred to higher levels of care, rather than being addressed at the community level, because of a lack of capacity.^5,6^

The University of Malawi began its specialised Masters in Family Medicine (FM) postgraduate training programme in 2015. The programme is offered by the Kamuzu University of Health Sciences Department of Family Medicine, with a vision of improving holistic community and district healthcare.^7^ Nkhoma Mission Hospital is one of the two rural hospitals used for FM training and serves a population of more than 450 000 people. Diamphwe, one of the rural HCs about 45 km away from Nkhoma, has a catchment of about 30 000 people. Although this HC often refers patients to Nkhoma Mission Hospital because of its proximity, its direct oversight and funding are through the District Health Office (DHO) under the Malawi Government Ministry of Health. At Diamphwe, two medical assistants, one laboratory technician and one pharmacy technician care for an average of 150 patients daily.

When FM registrars, based at Nkhoma Mission Hospital, began collaborating with the team at Diamphwe in 2019, there was no capacity to screen for or manage diabetes and hypertension. The only hypertension medications available included hydrochlorothiazide and propranolol; most first-line medications were not available because the HC did not have a clinical officer or access to appropriate laboratory testing.

## The intervention

The FM training programme at the Nkhoma Mission Hospital focused on existing HC resources and priorities in their partnership with Diamphwe to promote on-site ownership and long-term sustainability. A strong two-way communication set the foundation for collaboration with the HC, which was achieved through sequential site visits, as well as the creation of a WhatsApp group.

Initial discussions revealed a strong interest in NCD training and team capacity building at Diamphwe, which became the focus of the initial partnership. The feedback from Diamphwe staff was used to create continuing professional development on diabetes, hypertension and asthma, which was presented during site visits. Family medicine registrars worked with visiting undergraduate students from the Kamuzu University of Health Sciences and local staff to develop posters with dietary advice appropriate for the HC’s rural population. Members of the FM team also collaborated with local staff to develop clinic-appropriate protocols for diagnosing, managing and referring patients with diabetes and hypertension.

The lack of appropriate medication was one of the biggest challenges for increasing Diamphwe’s capacity to treat NCDs. One of the roles that the FM team played was advocating that the clinic should receive regular supplies of all essential NCD medicines from the DHO. Although Diamphwe’s lack of laboratory testing and advanced providers previously prevented it from qualifying for such medications, when the DHO became aware of the ongoing collaboration with FM at Nkhoma Mission Hospital, they authorised the medications.

**FIGURE 1:**
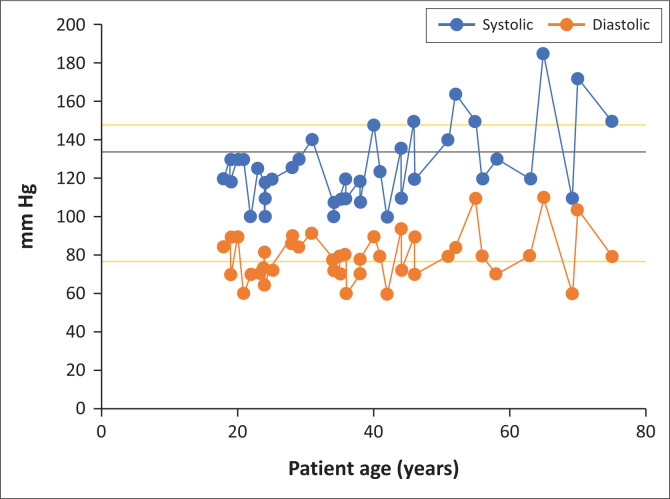
Blood pressure measurements in patients at Diamphwe health centres (*N* = 42).

Nkhoma Mission Hospital provided Diamphwe HC with electronic blood pressure (BP) machines and sent a team of FM undergraduate students to monitor the BP of 42 clinic patients during one of their site visits (Figure 1). Out of this small sample, 47% of those over the age of 40 years had hypertension, whilst only 4% of those under 40 years had reported hypertension. These data led the FM team to suggest that checking BP levels in all patients aged 40 years and older might detect about 90% of patients with hypertension (sensitivity 89%, specificity 73%).

This information was shared with the Diamphwe team, who decided to record and track individuals who presented with elevated BP or glucose levels in a register which was provided by the Nkhoma FM team, which also included appropriate treatment protocols. The blood glucose level was measured using a glucometer; glucose was measured in patients who were already diagnosed with hypertension and those who presented with symptoms of diabetes mellitus. Family medicine registrars continued to collaborate with the Diamphwe team through in-person visits and electronic communication for systems-level questions and individual patient consults. They also helped to support the Diamphwe team in requesting glucometers and supplies from the DHO.

## The effect on the community and health care services

The Diamphwe HC continues to log patients in the hypertension and diabetes registries and communicates with Nkhoma Mission Hospital FM registrars regarding difficult cases or refer patients. There is an ongoing agreement that the DHO will supply Diamphwe with all first-line hypertension as well as the diabetes medications, glibenclamide and metformin.

The Diamphwe pharmacy technician confirmed this in a recent interview, stating:

‘We have also learned a lot on how to use these medicines. Currently, we have about 50 patients that we have been following up with and managing their hypertension. We are very hopeful that more will register and be kept in the clinic’. (Male, 28 years old, pharmacy technician)

These sentiments were echoed by Diamphwe’s lead medical assistant:

‘Previously we did not have any medicines or glucometer for management of diabetes. Our confidence in managing these cases has increased and the fact that we can discuss cases with family medicine physicians any time on WhatsApp has helped a lot’. (Male, 40 years old, medical assistant)

Diamphwe’s laboratory technician provided a poignant assessment for the collaboration at the 20-month mark:

‘In general, our relationship with family physicians has improved the referral system of our patients. While in the past we would refer cases to the district hospital without discussing patients with specialists, nowadays we discuss most of the complicated cases with family physicians and some of the would-be-referred cases are effectively managed here following the discussions’. (Male, 25 years old, laboratory technician)

## The contribution of family medicine

Family medicine physicians and registrars have increased the capacity of Diamphwe HC to care for NCDs through teachings, collaborative communication and ongoing mentorship. Family medicine specialists have worked alongside the local team to develop relevant and utilisable protocols, clinic screening policies, case registries and patient education materials. This partnership provided an infrastructure, which helped the Diamphwe HC source medications to treat hypertension and diabetes within their community. This scalable, sustainable intervention helped to extend the impact of hospital-based FM physicians and improved care of NCDs within the community.

## Conclusion

A FM registrars-led collaboration rooted in communication and systems improvement has expanded the capacity of Diamphwe HC to care for patients with diabetes and hypertension. Site-driven, sustainable interventions have improved NCD patient care at the HC whilst providing residents with first-hand experiences in clinical leadership and quality improvement. Even as Diamphwe demonstrates how primary care facilities can improve NCD management, the FM registrars at Nkhoma Mission Hospital are distinguishing themselves as capable and efficient leaders of healthcare in Malawi.

## References

[1] Bickton FM, Lillie T. Strengthening human resources for health in resource-limited countries: The case of medic to medic in Malawi. Malawi Med J. 2019;31(1):99–101. 10.4314/mmj.v31i1.17 31143405 PMC6526336

[2] USAID. Global Health | Malawi | U.S. Agency for International Development [homepage on the Internet]. 2018 [cited 2021 May 6]. Available from: https://www.usaid.gov/malawi/global-health

[3] Makwero MT. Delivery of primary health care in Malawi. Afr J Prim Health Care Fam Med. 2018;10(1):a1799. 10.4102/phcfm.v10i1.1799 PMC6018651 29943590

[4] Government of the Republic of Malawi Health sector strategic plan II 2017–2022, towards universal health coverage. In: Ministry of Health Malawi Government, editor. 2nd ed. Zomba: National Statistics Office (NSO), 2017; p. 1–3.

[5] Msyamboza KP, Kathyola D, Dzowela T, Bowie C. The burden of hypertension and its risk factors in Malawi: Nationwide population-based STEPS survey. Int Health. 2012;4(4):246–252. 10.1016/j.inhe.2012.09.005 24029670

[6] Msyamboza KP, Mvula CJ, Kathyola D. Prevalence and correlates of diabetes mellitus in Malawi: Population-based national NCD STEPS survey. BMC Endocr Disord. 2014;14(1):41. 10.1186/1472-6823-14-41 24884894 PMC4018960

[7] Department of Family Medicine. Family medicine [homepage on the Internet]. School of Public Health and Family Medicine; 2016 [cited 2021 May 6]. Available from: http://sphfm.medcol.mw/family-medicine/

